# β-Cell protection and antidiabetic activities of *Crassocephalum crepidioides* (Asteraceae) Benth. S. Moore extract against alloxan-induced oxidative stress via regulation of apoptosis and reactive oxygen species (ROS)

**DOI:** 10.1186/s12906-017-1697-0

**Published:** 2017-03-29

**Authors:** Entaz Bahar, Kazi-Marjahan Akter, Geum-Hwa Lee, Hwa-Young Lee, Harun-Or Rashid, Min-Kyung Choi, Kashi Raj Bhattarai, Mir Mohammad Monir Hossain, Joushan Ara, Kishor Mazumder, Obayed Raihan, Han-Jung Chae, Hyonok Yoon

**Affiliations:** 1 College of Pharmacy, Research Institute of Pharmaceutical Sciences, Gyeongsang National University, Gyeongnam, Jinju, 52828 Republic of Korea; 2 Department of Pharmacology, Medical School, Chonbuk National University, Jeonju, 54896 Jeonbuk Republic of Korea; 3 Department of Pharmacy, University of Science and Technology Chittagong, Foy’s Lake, Chittagong, 4202 Bangladesh; 4 Department of Pharmacy, Jessore Science and Technology University, Jessore, 7408 Bangladesh

**Keywords:** *Crassocephalum crepidioides*, β-Cell Protection, Antidiabetes, Alloxan, Apoptosis, Reactive Oxygen Species, INS-1, Wistar Albino Rat

## Abstract

**Background:**

Medicinal plants are becoming more popular in the treatment of various diseases because of the adverse effects of the current therapy, especially antioxidant plant components such as phenols and flavonoids have a protective role against oxidative stress-induced degenerative diseases like diabetes. Thus, the purpose of this study was to investigate β-cell protection and antidiabetic activities of *Crassocephalum crepidioides* (Asteraceae) Benth. S. Moore.

**Method:**

The in-vitro study was conducted by the pancreatic β-cell culture and α-amylase inhibition technique which includes two methods, namely starch-iodine method and 3,5-dinitrosalicylic acid (DNSA) method. On the other hand, the in-vivo study was performed by oral glucose tolerance test (OGTT) method and alloxan-induced diabetes method by using Wistar albino rat. At the end pancreatic specimens were removed and processed for histopathological study.

**Result:**

The plant extract showed significant (*p < 0.05, **p < 0.01) effect on hyperglycemia as compared to standard (Gliclazide) in OGTT. The plant extract showed efficient protection activity of pancreatic β-cell from cell death in INS-1 cell line by significantly reduced (*p < 0.05, **p < 0.01) the levels alloxan-induced apoptosis and intracellular reactive oxygen species (ROS) accumulation. In addition, the plant extract showed a significant (*p < 0.05, **p < 0.01) effect on hyperglycemia by increases in percent of β-cells present in each islet (45% – 60%) compared to the diabetic group.

**Conclusion:**

The result showed that *C. crepidioides* had β-cell protection and antidiabetic activities in pancreatic β-cell culture and Wistar albino rat.

**Electronic supplementary material:**

The online version of this article (doi:10.1186/s12906-017-1697-0) contains supplementary material, which is available to authorized users.

## Background

Diabetes mellitus is a metabolic disorder characterized by high blood sugar (hyperglycemia) due to the disturbances of carbohydrate, fat and protein metabolism [1]. It can be classified as type-1 diabetes also known as insulin-dependent diabetes mellitus (IDDM) and type-2 diabetes also called non-insulin dependent diabetes mellitus (NIDDM) [2].

Pancreatic α-amylase inhibitors effectively reduced hyperglycemia through the control of starch breakdown [3]. Flavonoids or phenolic compounds containing plants can inhibit α-amylase enzyme lead to reduce blood glucose level [4]. Alloxan, is a potent diabetogenic agent that selectively damage pancreatic β-cells and it has been widely used as diabetes inducer [5]. It has been reported that alloxan rapidly and selectively accumulates in β-cells in comparison with non β-cells [6]. The alloxan-induced pancreatic β-cell damage through the generation of cytotoxic reactive oxygen species (ROS) [6–9]. The ROS produced by alloxan is responsible for the breakdown of DNA strand of pancreatic β–cells [10]. ROS induces oxidative stress that plays a pathological role in the development and progression of diabetic complications [11]. Thus, the identification of potent antidiabetic agents from natural sources, such as edible plants with minimum side effects, which will aid in the development of beneficial dietary guidelines for diabetic patients, is crucial. Recent studies have demonstrated that medicinal plants can protect β-cell by reducing alloxan-induced oxidative stress by scavenging ROS in-vitro and in-vivo, respectively [12–14].

The *Crassocephalum crepidioides* (*C. crepidioides*) Benth. S. Moore (Asteraceae) is an annual, edible herb also known as redflower ragleaf, that is widespread in many tropical and subtropical regions. The fleshy mucilaginous leaves and stems are commonly eaten and different parts of the plant have medical uses [15]. Moreover, it has been reported that *C. crepidioides* used to treat indigestion, stomach ache, epilepsy, sleeping sickness, swollen lips and it has also antitumor activity associated with nitric oxide production [16]. It is a potent antioxidant and protects against hepatotoxicity [17]. Our previous study found that, methanol extract of *C. crepidioides* possesses significant antioxidant activity as well as promising antihyperlipidemic activity [18]. Despite long traditional use of *C. crepidioides* as medicinal herb, no systemic phytochemical and pharmacological works have been carried out on this potential medicinal plant. Therefore, the aim of the present study to find out β-cell protection and antidiabetic effect *C. crepidioides*.

## Methods

### Plant material

The aerial parts of *C. crepidioides* was collected from Fatickchari, Chittagong in October 2013. The herbarium sheet was prepared by the expert following the standard procedure and taxonomic identification was made by Professor Shaikh Bokhtear Uddin, Department of Botany, University of Chittagong (CU), Bangladesh. A voucher specimen (Voucher number: SUS 5396) was deposited in Department of Botany, CU for preservation.

### Preparation of extract

The collected sampls were sliced into smaller pieces and dried at room temperature under shade and then powdered finely and subjected to extraction. Five hundred grams of the powder was macerated with about 2500 ml of 80% methanol for 15 days. The extract was then filtered and the marc was remacerated twice using the same volume of solvent to exhaustively extract the plant material [19]. The methanol was then removed from the extract by evaporation under reduced pressure using a rota vapor (BUCHI Rotavapour R-200, Switzerland) at 40 °C. The resulting dry hydroalcoholic extract was weighed and calculated for percentage yield, which was 23% (*w*/w). The dried plant extract was reconstituted with distilled water for oral administration.

### Identification of phenolic and flavonoids compound by chromatographic method

In order to recognize phenolic and flavonoids components of extract, column chromatography (CC) and thin layer chromatography (TLC) were used. The extract was subjected to CC with hexane: ethyl acetate in various solvent ratio. The fractions were collected depending on the visible changes in the colorful bands running out of the column. Then the fractions were subjected for TLC profiling. A variety of indicators, including iodine vapour, p-anisaldehyde-sulphoric acid, chloranil reagent, natural product reagent, aluminum chloride were used in this assay. The indicators were sprayed on TLC plate and observed at 254 nm and 365 nm wavelength under UV light. The R_f_ values resulted by test samples were 0.88 and 0.93 for extract which were parallel to the R_f_ values of reference compounds gallic acid and quercetin.

### Determination of α-amylase inhibition by starch-iodine color assay

Screening of plant material for α–amylase inhibitors was carried out in a microtitreplate according to based on the starch-iodine test [20]. The total assay mixture composed of 200 μL 0.02 M sodium phosphate buffer (pH 6.9 containing 6 mM sodium chloride), 60 μL of porcine pancreatic α–amylase (PPA) solution and plant extracts at concentration from 0 to 200 mg/mL (*w*/*v*) was incubated at 37 °C for 10 min. Then soluble starch 200 μL (1%, *w*/*v*) was added to each reaction well and the mixtures were then incubated at 37 °C for 15 min. The enzymatic reaction was stopped by the addition of 0.1 M HCl (120 μL) followed by the addition of 600 μL of iodine reagent (5 mM I_2_ and 5 mM KI). The color change was noted and the absorbance was read at 620 nm on a microplate reader. The control reaction representing 100% enzyme activity did not contain any plant extract. To eliminate the absorbance produced by plant extract, appropriate extract controls without the enzyme were also included. The known PPA inhibitor, acarbose, was used as positive control at a concentration range of 0–25 mg/mL. A dark-blue color indicated the presence of starch; a yellow color indicated the absence of starch while a brownish color indicated partially degraded starch in the reaction mixture. In the presence of inhibitors of the extracts, the starch added to the enzyme assay mixture was not degraded and gave a dark-blue color complex, whereas no color complex was developed in the absence of the inhibitor, indicating that starch was completely hydrolyzed by α–amylase.

### Determination of α-amylase inhibition by 3, 5-dinitrosalicylic acid (DNSA) assay

The inhibition assay was performed using the chromogenic 3, 5-dinitrosalicylic acid **(**DNSA) method [21]. The total assay mixture composed of 500 μL of 0.02 M sodium phosphate buffer (pH 6.9 containing 6 mM sodium chloride), 20 μL of PPA solution and extracts at concentration from 0 to 200 mg/mL (*w*/*v*) were incubated at 37 °C for 10 min. After pre-incubation, 500 μL of 1% (*v*/v) starch solution in the above buffer were added to each tube and incubated at 37 °C for 15 min. The reaction was terminated with 1.0 mL DNSA reagent, placed in boiling water bath for 5 min, cooled to room temperature, diluted and the absorbance measured at 540 nm. The known PPA inhibitor, acarbose was used a positive control at a concentration range of 0–25 mg/mL.

The IC_50_ values were determined from plots of percent inhibition versus inhibitor concentration and calculated by linear regression analysis from the mean inhibitory values. The IC_50_ values were defined as the concentration of the extract containing the α-amylase inhibitor that inhibited 50% of the PPA activity.

The other quantifiers were calculated as follows:

$$ \mathrm{Percentage}\ \mathrm{of}\ \mathrm{inhibition}\ \mathrm{of}\ \mathrm{the}\ \upalpha \hbox{--} \mathrm{amylase}\ \mathrm{activity}=\frac{\ \left(1-\mathrm{Abs}\left(\mathrm{control}\right)\right)\hbox{--} \left(1-\mathrm{Abs}\left(\mathrm{sample}\right)\ \right)}{1-\mathrm{Abs}\left(\mathrm{control}\right)}\mathrm{x}\ 100 $$

### Cell culture

INS-1, a rat insulinoma cell line, was obtained from the American type culture collection (ATCC), USA. The cells were grown in RPMI-1640 medium supplemented with 10% fetal bovine serum (FBS), 10 mmol/L HEPES, 2 mmol/L L-glutamine, 50 μmol/L β-mercaptoethanol, 1 mmol/L sodium pyruvate, 100 U/mL penicillin, and 100 μg/mL streptomycin at 37 °C in a humidified atmosphere containing 95% air and 5% CO_2_.

### Determination of cell viability

Cell viability was determined by crystal violet assay. Briefly, INS-1 cells were seeded onto 24-well plate (5 × 10^4^ cells/well), incubated overnight and pretreated with various concentrations of extracts (0–1000 μg/mL) for 24 h. Then, the medium was removed and cells were washed with phosphate buffer solution (PBS). 200 μL of 0.2% crystal violet solution was added to each well and incubated for 10 min at room temperature. Then wash with water and add 100 μL 1% SDS to solubilize the stain solution until color is uniform and no areas of dense coloration in bottom of wells. The samples were read at 590 nm in microplate reader (Spectra MAX, Gemini EM, Molecular Device).

### Analysis of the protective effect of the extract on oxidative stress induced cytotoxicity

Alloxan-induced cell survival was determined by crystal violet assay. Briefly, INS-1 cells were seeded (5 × 10^4^ cells/well) and cultured in 24-well culture plates. The cells were then preincubated with or without different concentrations of extract (25, 50, 100, and 200 μg/mL) at 37 °C in a humidified atmosphere of 5% CO_2_/95% air for 2 h followed by incubation with 200 μM alloxan for 24 h. After that the medium was removed and cells were washed with PBS and 200 μl of 0.2% crystal violet solution was added to each well and incubated for 10 min at room temperature. Then wash with water and add 100 μL 1% SDS to solubilize the stain solution until the color is uniform and no areas of dense coloration in bottom of wells. The samples were read at 590 nm in a microplate reader (Spectra MAX, Gemini EM, Molecular Device).

### Measurement of intracellular reactive oxygen species (ROS) level

ROS level was measured by using DCFH-DA method. DCFH-DA is a non-fluorescent compound, and it can be enzymatically converted to a highly fluorescent compound, DCF, in the presence of ROS [22]. In brief, after the treatment, INS-1 cells were washed with PBS and incubated with DCFH-DA at a final concentration of 10 μmol/L for 30 min at 37 °C in darkness. The fluorescence intensity was measured in the microplate reader (Spectra MAX, Gemini EM, Molecular Device) at an excitation wave length of 485 nm and an emission wave length of 538 nm after the cells were washed three times with PBS to remove the extracellular DCFH-DA. The level of intracellular ROS was showed as a percentage of non-treated control.

### Apoptosis assay

Hoechst33342 staining distinguishing apoptotic from normal cells based on nuclear chromatin condensation and fragmentation was used for the qualitative and quantitative analyses of the apoptotic cells. INS-1 cells were cultured in 6-well plates for 24 h. After treatment, the cells were incubated with 5 μg/ml Hoechst 33,342 for 15 min, washed twice with PBS, and then visualized by inverted fluorescence microscopy (Axioskop 2 plus microscope, Carl Zeiss, Oberkochen, Germany). The apoptotic nuclei were counted in at least 200 cells from five non overlapping fields in all treatment, and expressed as a percentage of the total number of nuclei counted.

### Experimental animal

Wistar albino rats of either sex, aged 8–10 weeks, weighing 120–250 g were used for the experiment. The animals were originally obtained from International Centre for Diarrheal Disease Research, Bangladesh (ICDDR, B). They were kept in clean and dry polypropylene cages with 12 h light, dark cycle at 25 ± 2 °C and 45–55% relative humidity in the animal house. The rats were fed with a standard laboratory diet and water ad libitum. Food was withdrawn 12 h prior to and during the experiment. As these animals are very sensitive to environmental changes, they are kept before the test for at least 3–4 days in the environment where the experiment will take place. The protocol used in this study was carried out with the guidelines of the Institutional Animal Ethics Committee (IAEC) and approval was taken from ethical committee of Faculty of Veterinary Medicine, Chittagong Veterinary and Animal Sciences University.

### Acute toxicity studies

Extract at the dose range of 100 mg to 2500 mg/kg were administered orally to different groups of rats, each group comprised of five rats. Mortality was observed after 72 h. Acute toxicity was determined according to the method of Litchfield and Wilcoxon [23].

### Oral glucose tolerance test (OGTT)

OGTT is one of the most acceptable methods to evaluate the hypoglycemic activity [24]. Twenty fasted rats were divided into four groups with each group containing five rats. Group 1 was kept as control which received vehicle orally (p.o.), group 2 received gliclazide 10 mg/kg as standard, and group 3 and 4 received extracts 300 mg/kg and 500 mg/kg, respectively. After 30 min of extracts and gliclazide administration all groups were orally treated with 3 g/kg of glucose. The blood glucose level was measured by glucometer using glucose estimation kit at 0, 30, 90 and 120 mins of glucose loading. Blood samples were collected from the tail vein.

### Induction of diabetes in experimental animals

The rats were fasted for 12 h prior to the induction of diabetes. Alloxan was freshly prepared in 0.2 ml 0.9% normal saline was administered intraperitoneally (i.p.) at a single dose of 150 mg/kg. Development of diabetes was confirmed by measuring the blood glucose concentration at 48 h after the administration of alloxan. Rats with blood glucose levels of <7 mmol/dL were excluded from the experiment. Treatment with plant extracts was started 48 h after alloxan injection [25].

### Experimental design

Animals were divided into five groups with each group receiving solutions as follows:

1. Normal control (Saline 10 mL/kg, p.o.)
2. Alloxan treated control (150 mg/kg, i.p.)
3. Alloxan (150 mg/kg, i.p.) + plant extract (150 mg/kg, p.o.)
4. Alloxan (150 mg/kg, i.p.) + plant extract (300 mg/kg, p.o.)

Saline and plant extract (150 and 300 mg/kg) were administered to diabetic rats for 14 days and the blood glucose level was measured at 0, 3, 5, 7, 9 and 14th days. After 14 days of plant extract treatment, the animals were sacrificed and blood was collected, sera separated by centrifugation at 3 000 *g* for 10 min. Details of the estimation of insulin, urea, creatinine and glycosylated haemoglobin are given in Additional file 1.

### Haemetoxylin and eosin staining

After plant extract treatment, pancreas from the normal control, alloxan control, and plant extracts (150 and 300 mg/kg)-treated rats were quickly removed for histological studies. Removed pancreatic tissues were washed in saline, fixed in 10% formalin and fixed10% neutral buffered formalin (NBF) for 48 h and processed with paraffin embedding. The sections stained in 0.1% Mayer’s haemetoxylin, counterstained in 0.5% eosin, mounted and were observed under microscope.

### Statistical data analysis

All the data were expressed as mean ± SD and one way ANOVA (Analysis of variance) followed by Dunnett’s test was used for the statistical analysis using SPSS software (version 16). *p < 0.05 and **p < 0.01 were considered significant.

## Results

### Phenols and flavonoids

Phytochemical assay by chromatographic method showed that the main components, including phenolic and flavonoid compounds were present in the extract (Table 1).

**Table 1:** Phytochemical result of extract

Compounds	Reagents	Standards	Results
Phenols	P-Anisaldehyde-Sulphoric acid	Gallic acid	+
	Chloranil reagent	Gallic acid	+
Flavonoids	Natural product reagent	Quercetin	+
	Aluminum chloride	Quercetin	+

### α-Amylase inhibition activity

**Fig. 1:**
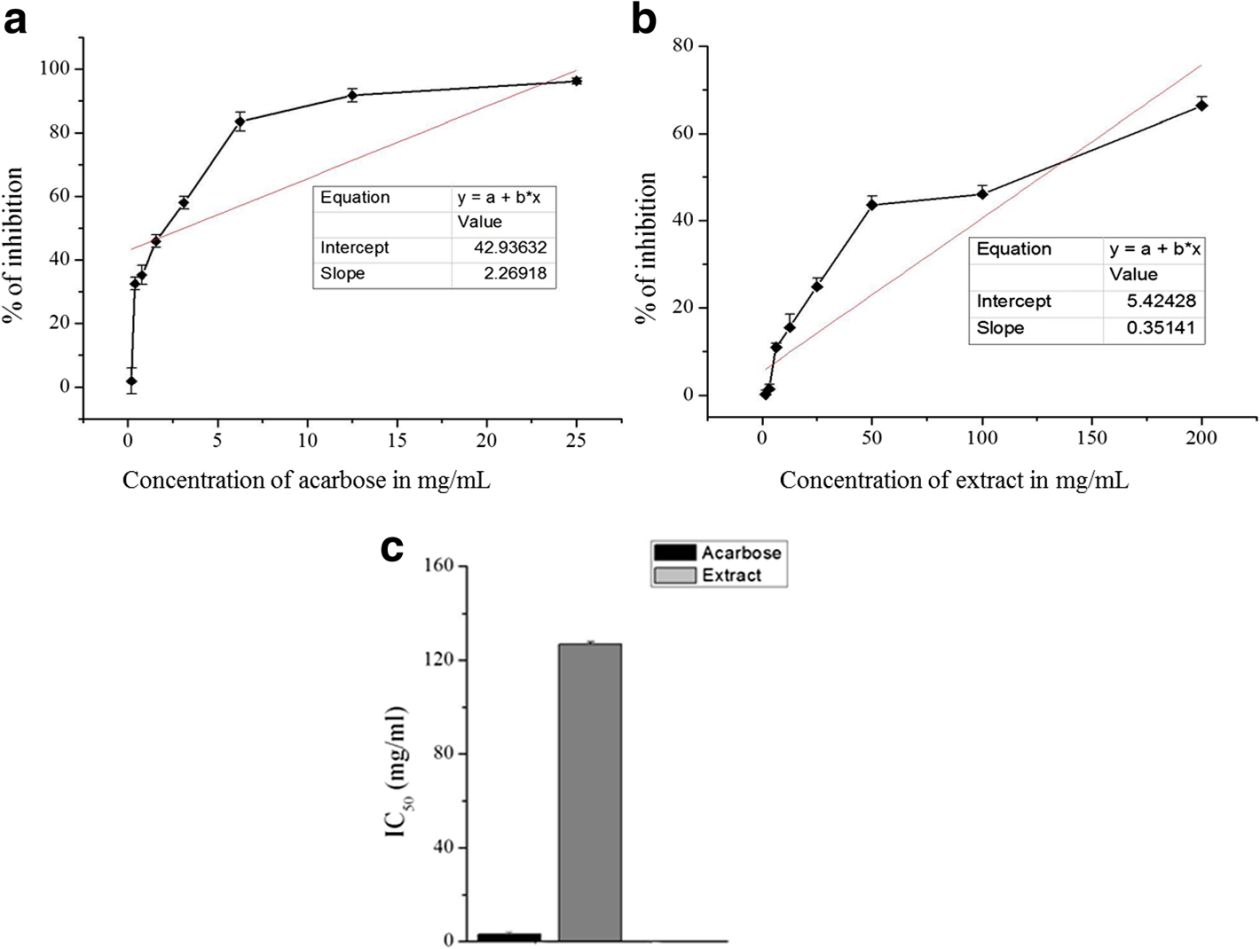
α-amylase inhibition activity of acarbose and *C. crepidioides* in starch-iodine assay. **a** Percentage inhibition of acarbose (**b**) 80% methanol extract of *C. crepidioides* and (**c**) IC_50_ of treatment groups on α -amylase enzyme in vitro. Values were represented as mean ± SD (*n* = 3)

**Fig. 2:**
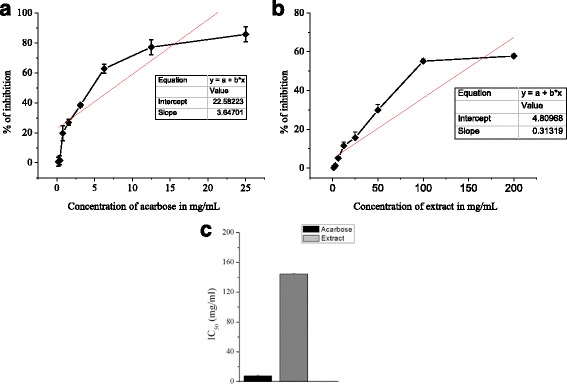
α-amylase inhibition activity of acarbose and *C. crepidioides* in DNSA assay. **a** Percentage inhibition of acarbose (**b**) 80% methanol extract of *C. crepidioides* and (**c**) IC50 of treatment groups on α -amylase enzyme in vitro. Values were represented as mean ± SD (*n* = 3)

In the present study of the in-vitro α-amylase inhibition activity test, the extract showed significant % inhibition of α-amylase relative to the standard treatment, acarbose in starch-iodine test and DNSA assay, respectively (Figs. 1 and 2). In starch-iodine test, acarbose showed highest α-amylase inhibition of 96.24% at a concentration of 25 mg/mL (Fig. 1a) with an IC50 value 3.11 ± 0.10 mg/mL (Fig. 1c), while the extract showed a highest α-amylase inhibition of 66.37% at a concentration of 200 mg/mL (Fig. 1b) with an 126.85 ± 2.10 mg/mL (Fig. 1c). In DNSA assay, acarbose showed highest inhibition of 85.63% at a concentration of 25 mg/mL (Fig. 2a) with an IC_50_ value 7.52 ± 0.30 mg/mL (Fig. 2c), while the extract produced a highest inhibition of 57.76% at a concentration of 200 mg/mL (Fig. 2b) with an IC_50_ value 144.29 ± 5.10 mg/mL (Fig. 2c).

### Effect of extract on INS-1 cell lines

**Fig. 3:**
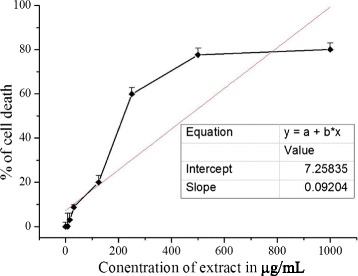
Concentration dependent effect of *C. crepidioides* extract on INS-1 cell line. Results are expressed as % of cell reduction (= % of cell death). Values were represented as mean ± SD (*n* = 4). Toxicity results for INS-1 cell line incubated with different concentrations of extract

The cytotoxic effect of the extract on rat pancreatic cell line INS-1 was evaluated by incubating it with various concentrations of extract (1–1000 μg/mL). The toxicity results revealed a decrease in percentage of viability at higher concentrations of the extract and the IC_50_ value was found to be 464.38 ± 3.1 μg/mL (Fig. 3).

### Protective effect of extract on alloxan induced cytotoxicity

**Fig. 4:**
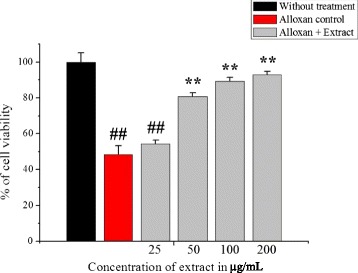
Protective effect of *C. crepidioides* extract on alloxan induced cell cytotoxicity in INS-1 cells. Values were represented as mean ± SD (*n* = 4). ^##^ *p* < 0.01 as compared with the control group; **p < 0.01 as compared with the alloxan control group. Toxicity results of INS-1 cell line induced with 200 μM of alloxan and preincubated with different concentrations of extract

The effect of extract on the viability of INS-1 cells cultured under alloxan-induced toxicity conditions was measured by crystal violet assay. Pretreatment of INS-1 cells for 2 h with extract at concentrations of 25–200 μg/mL significantly protected INS-1 cells from alloxan toxicity. An increase in cell viability was observed in treated cells compared to alloxan control in a dose dependent manner (Fig. 4). The result displayed that extracts (50 μg/mL, 100 μg/mL and 200 μg/mL) possessed the best protective effects (*p* < 0.01).

### Effect of extract on intracellular ROS activity

**Fig. 5:**
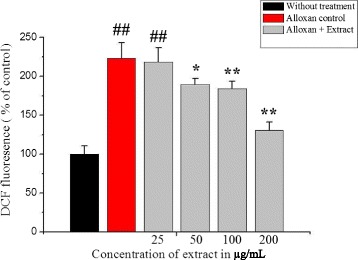
Protective Effect of *C. crepidioides* extract on alloxan-induced ROS in INS-1 cells. DCF fluorescence reflects ROS level. Values were represented as mean ± SD from three independent experiments. ^##^ *p* < 0.01 as compared with the control group; *p < 0.05 and **p < 0.01 as compared with the alloxan control group. Intracellular ROS activity results for INS-1 cell line induced with 200 μM of alloxan and pre incubated with different concentrations of extract

As shown in Fig. 5, after exposed to 200 μmol/L alloxan for 24 h, the intracellular ROS level of the INS-1 cells markedly increased to 223.36% relative to the control value (100%, *p* < 0.01), which suggests that alloxan induce oxidative stress [26, 27]. When the cells were incubated with different concentrations of extract (25, 50, 100 and 200 μg/mL) in the presence of 200 μmol/L alloxan for 24 h, the intracellular ROS levels significantly decreased to 218.22%, 189.73%, 184.07% and 130.8% of the control value (100%, *p* < 0.01), respectively.

### Effect of the extract on alloxan-induced apoptosis in INS-1 cells

**Fig. 6:**
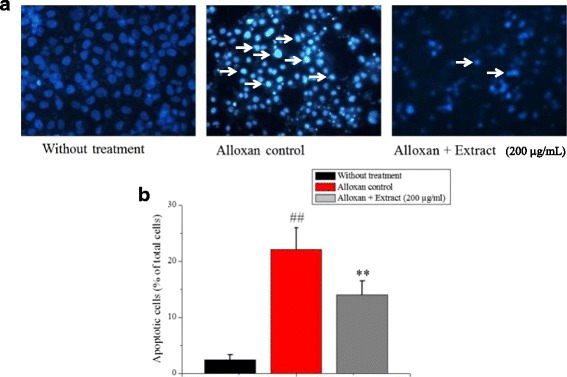
Protective effects of *C. crepidioides* extract on the cell survival in alloxan-treated INS-1 cells by Hoechst 33,342 staining method. **a** Representative images by Hoechst 33,342 staining, arrowheads in the pictures indicate the nuclei of the apoptotic cells (Hoechst-positive cells). **b** The apoptosis rate was determined by calculating the percentage of Hoechst positive cells over the total number of cells. Values were represented as mean ± SD from three independent experiments. ^##^ *p* < 0.01 as compared with the control group; **p < 0.01 as compared with the alloxan control group. Apoptosis activity results for INS-1 cell line induced with 200 μM of alloxan and pre incubated with different concentrations of extract

INS-1 cells treated with 200 μmol/L for 24 h showed typical characteristics of apoptosis, including the condensation of chromatin, the shrinkage of nuclei, alteration of size, shape and number of nuclei using Hoechst 33,342 staining as shown in (Fig. 6a). The amount of apoptotic nuclei was markedly increased, and the apoptosis rate increased to 22.10% relative to the control group (100%, *p* < 0.01). However, pretreatment with extract (200 μg/ml) in the presence of alloxan (200 μmol/L), the number of apoptotic cells was obviously decreased, and the apoptosis rate reduced to 14.81% relative to the control group (100%, *p* < 0.01) (Fig. 6b).

### Acute toxicity

The plant extract did not show any sign of acute toxicity up to a dose of 2500 mg/kg of body weight. The behavior of the animals was clearly observed for the first 8 h, then at an interval of every 8 h during next 72 h*.* The extract did not produce a significant change in animal behavior or mortality.

### Effect on OGTT

**Fig. 7:**
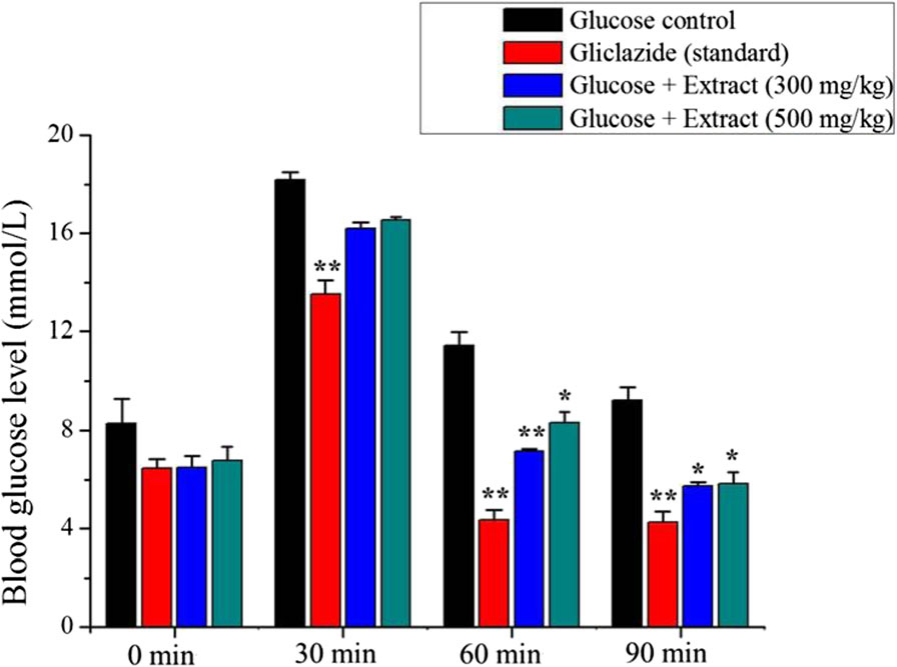
Effect of *C. crepidioides* extract on OGTT. Values were represented as mean ± SD (*n* = 6). *p < 0.05 and **p < 0.01 as compared with the glucose control group

It was observed that the plant extracts showed a significant effect on hyperglycemia as compared to standard treatment, gliclazide (Fig. 7). In the glucose control rats, the blood glucose level was increased after 30 min and remained high over the next 120 min. Plant extracts (300 mg/kg and 500 mg/kg) significantly reduced (*p* < 0.05) the blood glucose level at 30 min and remain low over the next 120 min when compared with the glucose control.

### Effect on alloxan-induced diabetes

**Fig. 8:**
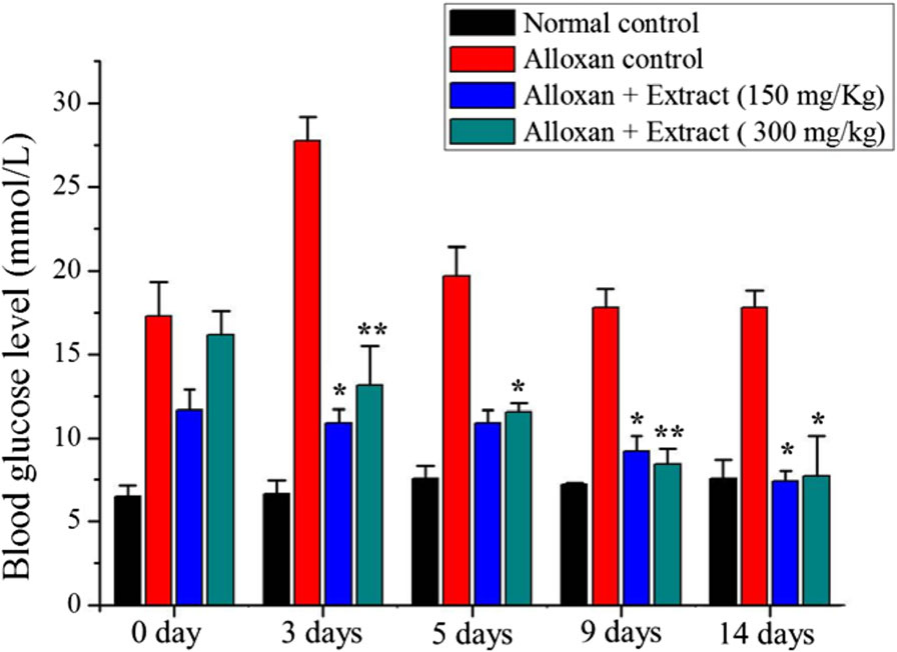
Effect of *C. crepidioides* extract on alloxan-induced diabetic rats. Values were represented as mean ± SD (*n* = 6). *p < 0.05 and **p < 0.01 as compared with the alloxan control group

It was observed that both doses (150 mg/kg and 300 mg/kg) of the extract showed successful hypoglycemic activity (Fig. 8). In the diabetic control rats, the blood glucose level was increased and remained high over the 14th days. Plant extracts significantly reduced (*p* < 0.05) the blood glucose level when compared with the diabetic control.

### Morphological studies

**Fig. 9:**
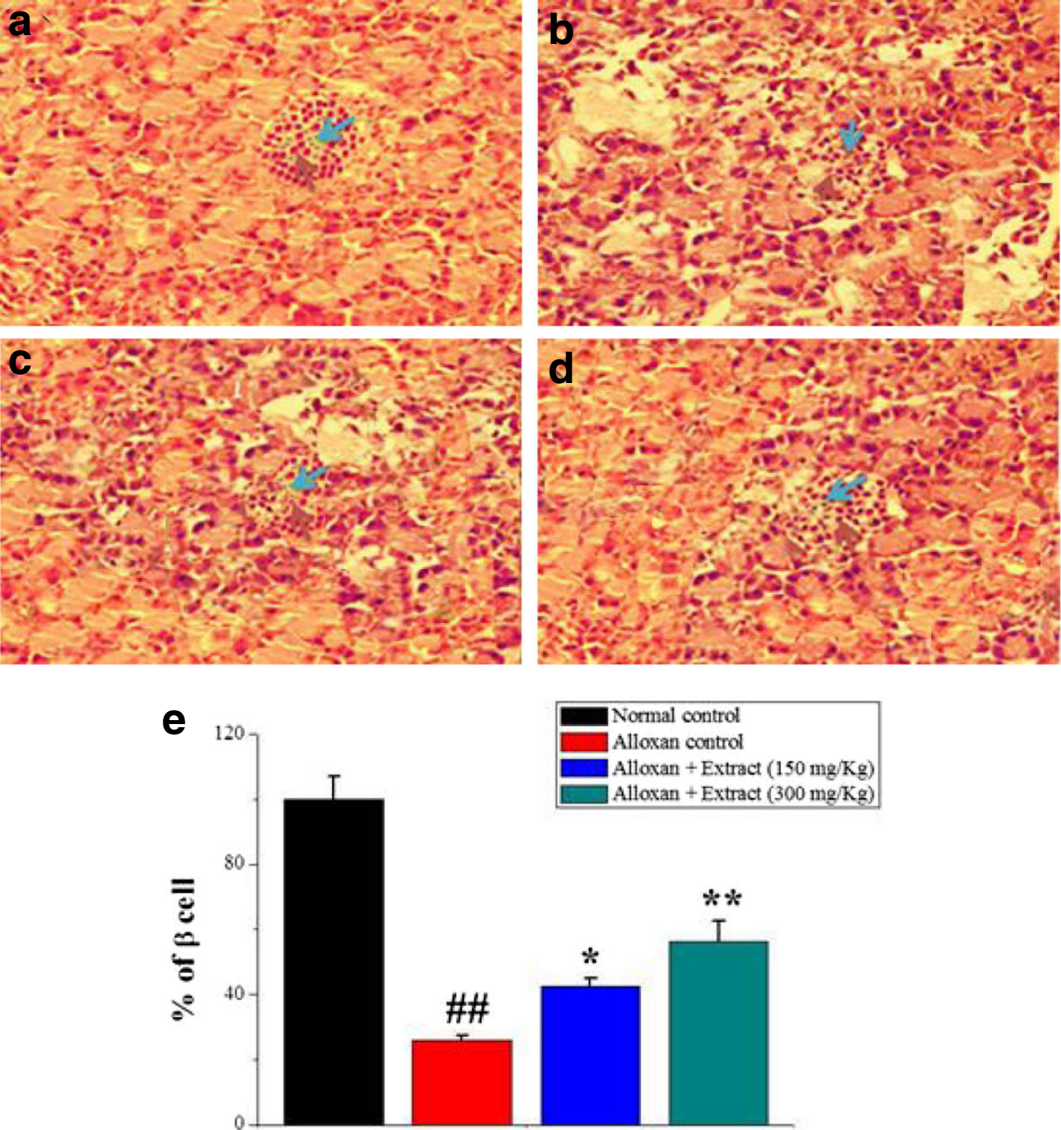
Histopathological examination under microscopy: (**a**) Normal cellular population in the islets of langerhans in pancreas of vehicle-treated rats. **b** Extensive damage to the islets of langerhans and reduced dimensions of islets in diabetic control rats. (**c**, **d**) The partial restoration of normal cellular population and enlarged size of β-cells were shown by the extracts (150 mg/kg and 300 mg/kg), respectively. **e** The β-cell regeneration rate was determined by calculating the percentage of H & E positive cells over the total number of cells. Values were represented as mean ± SD. ^##^ *p* < 0.01 as compared with the control group; *p < 0.05 and **p < 0.01 as compared with the alloxan control group

In histopathological examination, rat pancreatic tissue of the normal group showed regular exocrine tissue with interspersed islets of langerhans. No cellular abnormality or inflammatory infiltration was seen (Fig. 9a). The rat pancreatic tissue of the diabetic control group revealed regular exocrine pancreas with a reduced number of islets of langerhans. As a whole, each islet contained a reduced number of cells. Furthermore, few pyknotic cells along with mild infiltration of lymphocytes were observed within the islets (Fig. 9b). Rat pancreatic tissues of the plant extract 150 mg/kg and 300 mg/kg groups showed regular exocrine pancreas with moderately increased number and size of islets of langerhans as well as increased number of cells per islets with a distinct margin between exocrine and endocrine tissue (Fig. 9c, d). In the plant extract-treated group, morphometric results showed a significant increase in the number of the islets/mm^2^ (Table 2) and the number of β-cells in islet compared to the diabetic group (Table 3). There was no significant difference in the number of islets/mm^2^compared to the normal group. There was a significant decrease in the percent of β-cells (25% – 30%) in the diabetic group, while there was a significant increase in the plant extract-treated group (45% – 60%) compared to the diabetic groups (Fig. 9e).

**Table 2:** The number of islets/mm^2^ in the control and treatment groups

Groups	Number of islets/mm^2^
Normal control	5.2 ± 0.62
Alloxan control	1.4^##^ ± 0.93
Alloxan + Extract (150 mg/kg)	2.9^*^ ± 0.44
Alloxan + Extract (300 mg/kg)	3.9^**^ ± 0.31

Values were represented as mean ± SD. ^##^ *p* < 0.01 as compared with the control group; *p < 0.05 and **p < 0.01 as compared with the alloxan control group

**Table 3:** The number of β cells per islet in the control and treatment groups

Groups	Number of β cells/ islet
Normal control	94.60 ± 6.02
Alloxan control	28.92^##^ ± 2.13
Alloxan + Extract (150 mg/kg)	42.2^*^ ± 3.31
Alloxan + Extract (300 mg/kg)	56.4^**^ ± 4.01

Values were represented as mean ± SD. ^##^ *p* < 0.01 as compared with the control group;

*p < 0.05 and **p < 0.01 as compared with the alloxan control group

## Discussion

The blood glucose level has been increased by the α-amylase and α-glucosidase enzyme which break carbohydrate to simple absorbable monosaccharides [28]. The α-amylase and α–glucosidase inhibitors have been useful as oral hypoglycemic drugs for the control of hyperglycemia especially in patients with type II diabetes mellitus [29]. Inhibition of α-amylase delays carbohydrate digestion and prolong overall digestion time lead to reduce rate of glucose absorption and ultimately reducing the blood glucose level [30]. In our study, the plant extract showed significant % inhibition of α-amylase as compared to the standard anti-diabetic drug, acarbose, in both starch-iodine test and DNSA assay that could be attributed to the presence of phenolic and flavonoids components (Table 1) because polyphenols are capable of inhibiting carbohydrate hydrolyzing enzymes due to their ability to bind with proteins [31, 32].

Our results in this study showed pretreatment of INS-1 cells cultured under alloxan-induced cytotoxicity, with extract significantly increased survival and decreased rate of cell death. Treatment of INS-1 cells with extract also reduced ROS intracellular formation and decreased cell apoptosis that indicate the pancreatic β-cell protective effect of extract on the INS-1 cell line [33–35].

Furthermore, the study revealed that the plant extract showed a significant effect on hyperglycemia as compared to standard treatment, gliclazide, in OGTT. OGTT was used to identify the altered carbohydrate metabolism during post glucose administration. The ability of extract to lower the blood glucose level in oral glucose tolerance test suggests that rats treated with plant extract had better glucose utilization capacity [36]*.* It is also well established that sulfonylureas (e.g. gliclazide) cause hypoglycemia by stimulating insulin release from pancreatic β-cells [37]. Our result showed that the extract groups of diabetic rats significantly increased (*p* < 0.05 or *p* < 0.01) the serum insulin (Additional file 1: Table S1). The comparable effect of the extract with gliclazide suggests the possibility of a similar mode of action.

Additionally, our results demonstrated that the plant extracts showed significant effect on hyperglycemia as compared to alloxan control rats. The blood glucose lowering effect of plant extract could be attributed to the presence of flavonoids and phenolic compounds (Table 1) that have been associated with hypoglycemic activity [38, 39].

**Fig. 10:**
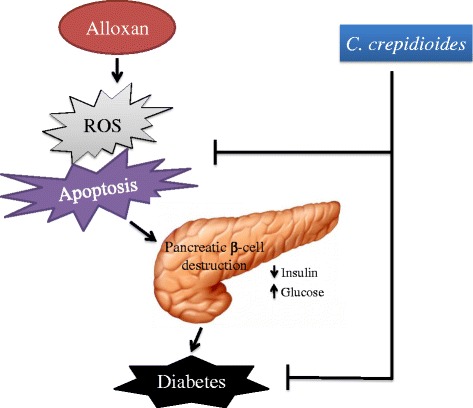
Proposed mechanism of β-cell protection and antidiabetic activities of *C. crepidioides* against alloxan-induced oxidative stress. The schematic diagram showing the proposed mechanism underlying the β-cell protection and antidiabetic effect of *C. crepidioides* against the alloxan-induced ROS production, apoptotic degeneration and pancreatic destruction

Histological findings of the pancreatic tissue using haematoxylin of the control group showed bluish-stained β-cells filling the interior of the islet, while pink-stained α-cells were arranged in mainly clusters located at the periphery of the islets and also between β-cells. Alloxan administration exhibited degenerative changes at the center of islets of langerhans and also decreased number of the bluish-stained β-cells, while the pink-stained α-cells remained present at the periphery of the islet. The alloxan caused significant morphological changes in diabetic rats with severe pancreatic β-cells destruction, such as decreasing the islets cell numbers, cell damage, and cell death as compared to the control group [40, 41]. The cytotoxic action of alloxan is mediated by ROS, with a simultaneous massive increase in cytosolic calcium concentration leading to rapid destruction of β-cells [42, 43]. The plant extract showed a significant increase in the number of the islets/mm^2^ and the number of β-cells in islet compared to diabetic group. The plant extract may act on islet functions (Table 2) lead to increasing the number of pancreatic islets (Table 3) pancreatic β-cells from oxidative damage through regulating ROS production (Fig. 5) and apoptotic rate (Fig. 6) [44, 45]. In summary, the results of this study showed the pancreatic β-cell protective properties of the extract through reducing the ROS production and decreasing the cell apoptosis (Fig. 10) that might be due to presence of flavonoids, polyphenolic compounds (Table 1) [46]. Therefore, this medicinal plant as a Complementary Medicine strategy may be used in metabolic diabetic diseases; however, further study is needed.

## Conclusions

This study demonstrates that the extract possesses a significant anti-diabetic activity at different dose levels in both in-vitro and animal models. Hence, the study of in-vitro and in-vivo pharmacological and phytochemical evaluation of this plant forms a primary platform for future studies. The anti-apoptotic signaling mechanism of active compounds from this promissory extract should be further investigated as a natural remedy for diabetes treatment.

## Additional file

Additional file 1: Effect of *C. crepidioides* on the serum insulin, urea, creatinine and HbA1c. (DOC 41 kb)

## Abbreviations

DNSA: 5-dinitrosalicylic acid
h: Hour
IC_50_: 50% inhibitory concentration
kg: Kilogram
mg: Milligram
min: Minute
mL: Milliliter
OGTT: oRal glucose tolerance test
PBS: Phosphate buffer solution
PPA: Porcine pancreatic α-amylase
ROS: Reactive oxygen species
SD: Standard deviation
SDS: Sodium dodecyl sulfate
TLC: Layer chromatography
UV: Ultraviolet
μg: Microgram
