# Supplementary material for: β-Cell protection and antidiabetic activities of Crassocephalum crepidioides (Asteraceae) Benth. S. Moore extract against alloxan-induced oxidative stress via regulation of apoptosis and reactive oxygen species (ROS)
Source: BMC Complement Altern Med. 2017 Mar 29;17:179. doi: 10.1186/s12906-017-1697-0 (PMC5372275; doi:10.1186/s12906-017-1697-0)
Supplement: Additional file 1: — Effect of C. crepidioides on the serum insulin, urea, creatinine and HbA1c. (DOC 41 kb) [file 12906_2017_1697_MOESM1_ESM.doc]

Additional file 1

Materials and Methods

**Estimation of insulin, urea, creatinine and glycosylated haemoglobin**

After fourteen days of treatment, the animals were sacrificed and blood was collected, sera separated by centrifugation at 3 000 g for 10 min. Insulin level was assayed by ELISA kit . Estimation of urea, serum creatinine and glycosylated haemoglobin (HbA1c) were carried out by the previously described method .

Results

**Effect of extracts on the serum insulin, urea, creatinine and HbA1c level rats**

# Serum insulin level of alloxan control group was significantly (**P**<0.01) decreased when compared to normal control group. The extract groups of diabetic rats significantly increased (p <0.05 or **P**<0.01) the serum insulin. A significant (P<0.01)) elevation in urea and creatinine was observed in alloxan-induced diabetic rats when compared to normal control rats. The extracts were administrated orally to diabetic rats for 14 days reversed the urea and creatinine level to near normal. Administration extract significantly (**P**<0.05 or P <0.01) reduced HbA1c level compared to diabetic control rats (Table S1).

# Table S1: Effect of *C. crepidioides* on the serum insulin, urea, creatinine and HbA1c.

| Treatment groups | Insulin (mIU/mL) | Urea (mg/dL) | Creatinine (mg/dL) | HbA1c (%) |
| --- | --- | --- | --- | --- |
| Normal control | 34.435±2.30 | 16.13±1.44 | 0.63±0.02 | 3.04±0.32 |
| Alloxan control | 11.345±1.01^##^ | 31.42±2.85^##^ | 4.45±0.59^##^ | 11.34±0.78^##^ |
| Alloxan + Extract (150 mg/kg) | 18.432±1.990^*^ | 25.76±2.26^*^ | 2.46±0.48^*^ | 8.26±0.76^*^ |
| Alloxan + Extract (300 mg/kg) | 25.940±2.090^**^ | 18.32±1.06^**^ | 1.15±0.11^**^ | 5.54±0.62^**^ |

Values are expressed as mean±SD (n=5). ##*p* < 0.01, compared to the control group;
**p* < 0.05; ***p* < 0.01, compared to the alloxan group.

1. Andersen L, Dinesen B, Jorgensen PN, Poulsen F, Roder ME: **Enzyme immunoassay for intact human insulin in serum or plasma**. *Clinical chemistry* 1993, **39**(4):578-582.

2. Owen JA, Iggo B, Scandrett FJ, Stewart CP: **The determination of creatinine in plasma or serum, and in urine; a critical examination**. *The Biochemical journal* 1954, **58**(3):426-437.

3. Balamurugan K, Nishanthini A, Mohan VR: **Antidiabetic and antihyperlipidaemic activity of ethanol extract of Melastoma malabathricum Linn. leaf in alloxan induced diabetic rats**. *Asian Pacific journal of tropical biomedicine* 2014, **4**(Suppl 1):S442-448.

4. Karunanayake EH, Chandrasekharan NV: **An evaluation of a colorimetric procedure for the estimation of glycosylated haemoglobin and establishment of reference values for Sri Lanka**. *J Natl Sci Counc Sri Lanka* 1985, **193**:235–258.
